# Veno-arterial extracorporeal membrane oxygenation for post-infarction ventricular septal defect in a low-volume center

**DOI:** 10.1051/ject/2023013

**Published:** 2023-09-08

**Authors:** Miha Antonic, Anze Djordjevic, Tomaz Podlesnikar, Maja Pirnat, Boris Robic, Rene Petrovic, Igor D. Gregoric

**Affiliations:** 1 Department of Cardiac Surgery, University Medical Centre Maribor Ljubljanska ulica 5 2000 Maribor Slovenia; 2 Department of Radiology, University Medical Centre Maribor Ljubljanska ulica 5 2000 Maribor Slovenia; 3 Center for Advanced Heart Failure, Cardiopulmonary Support and Transplantation Program, Memorial Hermann Heart & Vascular Institute - Texas Medical Center Fannin Street 6400 77030 Houston Texas USA

**Keywords:** Ventricular septal defect, Cardiogenic shock, Extracorporeal membrane oxygenation, Mediastinitis, ECMO

## Abstract

Managing patients with post-ischaemic ventricular septal defects (VSD) and postcardiotomy cardiogenic shock can be extremely challenging in a low-volume cardiac surgery unit. We present a case of a 68-year-old patient who received veno-arterial extracorporeal membrane oxygenation support due to cardiogenic shock after VSD repair. The patient was successfully weaned off support after 86 h. In the postoperative period, mediastinitis occurred, and negative pressure wound therapy was instituted.

## Overview

There has been an exponential rise in the use of extracorporeal membrane oxygenation (ECMO) therapy in various clinical settings, including postcardiotomy cardiogenic shock (PCCS). Survival of these patients has only slightly improved during the last two decades despite important technological advances, such as using last-generation oxygenators, magnetic-drive centrifugal pumps, and heparin-covered circuits. The mortality in adult ECMO is still very high, with only 30–35% of patients surviving until hospital discharge [1].

Despite some heterogeneity in the definition, the term PCCS has been used to describe cardiogenic shock occurring from 0 to 48 h after separation from cardiopulmonary bypass (CPB). The incidence of PCCS is reported to be 1.0% to 1.4% of patients undergoing CPB. Complications while on ECMO support are frequent and include bleeding, renal failure, infection, and neurological and gastrointestinal complications [2–6]. Due to the relatively small number of these patients, low-volume cardiac surgery centers may lack experience with temporary mechanical circulatory support (MCS). Our cardiac center performs 400–500 open heart procedures and 20–30 ECMO cannulations annually. Like many hospital centers in the US, our Slovene system is small, and distance and access limit the ability to transfer patients in emergent cases. Thus, we present a patient with PCCS after repair of a post-infarction ventricular septal defect (VSD), who was successfully treated with ECMO at a low-volume cardiac center.

## Description

**Figure 1:**
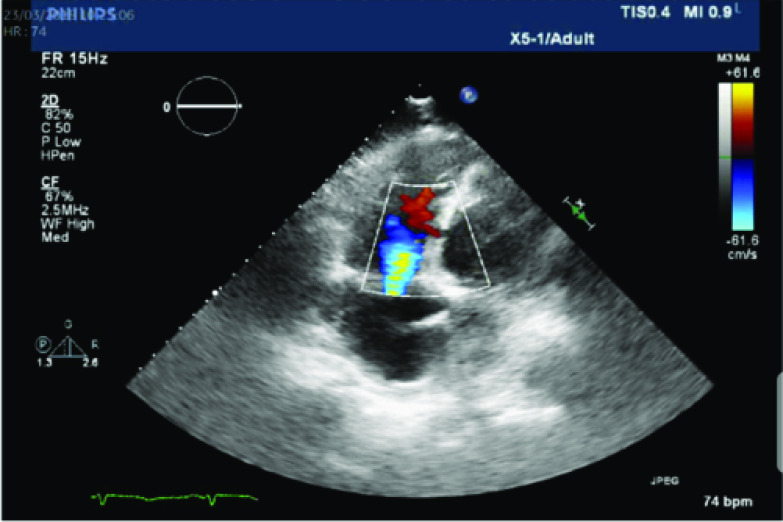
A large posterobasal ventricular septal defect as seen on transthoracic echocardiography.

Written patient consent was obtained. A 68-year-old Caucasian male was admitted to the hospital due to borderline low blood pressure (112/76 mm Hg), shortness of breath, and nausea in the five days before admission. Transthoracic echocardiography revealed a large posterobasal VSD with a significant left-to-right shunt. The diameter of the VSD was 2 cm, the maximal pressure gradient was 40 mmHg, and the ratio Qp/Qs was 2.5 (Figure 1). In addition, an aneurysm of the inferior wall and severe tricuspid valve regurgitation were noticed. Coronary angiography revealed an occluded right coronary artery. No primary coronary intervention (PCI) attempts were made due to the delayed presentation and existing VSD and posterior aneurysm. An intra-aortic balloon pump (IABP) was immediately inserted to delay surgery for at least ten days.

The patient became hemodynamically unstable six days after IABP placement. In consultation with the only other ECMO center in our country, the decision was made to emergently transfer the patient to the operating room. The VSD was closed using the double-patch technique [7]. In addition, the posterior wall aneurysm was repaired, and a tricuspid valve annuloplasty was performed using the 32-size Edwards Physio Tricuspid Ring (Edwards Lifesciences), with additional bicuspidalization of the tricuspid valve. Total CPB time was 296 min, aortic cross-clamp time was 172 min, and on-pump urinary output was 530 cc. After separation from CPB, the low-output syndrome was observed with an estimated left ventricular ejection fraction (LVEF) < 10%. The patient was centrally cannulated for veno-arterial ECMO (V-A ECMO), with venting of the left ventricle (LV) through the right superior pulmonary vein. After 24 h, the patient’s hemodynamic parameters improved (LVEF of 20–25% and no regional wall motion abnormalities). ECMO support was switched to peripheral cannulation. Our institution follows the short-term MCS weaning protocol as previously described [8]. As the patient’s end-organ function was satisfactory, the LVEF was >25%, and the patient tolerated vasoactive support, the vent and the IABP were removed, and the chest was closed. After 86 h of ECMO support, the LVEF improved to 30%, and there was no ventricular dilation nor mitral or tricuspid regurgitation. The patient had a central venous pressure of 12 mm Hg and oxygen saturation of 95% on FiO_2_ 50%. He required moderate vasoactive support with 0.10 mcg/kg/min of norepinephrine. The patient was decannulated from ECMO support.

He was discharged from the intensive care unit on postoperative day 12. After multiple thoracenteses for pleural effusions and slow rehabilitation, purulent discharge was noticed on the sternotomy wound. Computer tomography images revealed severe mediastinitis. Osteosynthetic material (four sternal stainless-steel wires) was removed, and negative pressure wound therapy was instituted for 23 days, as previously described [9]. He was discharged home on postoperative day 65.

## Comment

The mortality rate of postoperative cardiogenic shock remains high [1, 10–12]. Based on data from smaller, contemporary studies, the rate has possibly improved due to the evolution of ECMO technology over the last two decades and the innovations of MCS devices [13, 14]. Cardiogenic shock still represents a challenging spectrum of cardiac surgery, and smaller cardiac centers find it difficult to manage this patient population. This case report encourages smaller centers to use MCS more aggressively for similar cases and displays the feasibility of successfully managing these patients.

Proper patient selection for temporary MCS is crucial. As reported by Khorsandi et al. and Schaefer et al., age is one of the most significant predictors of worse outcomes, and patients above 70 years old have a higher mortality risk if put on ECMO [1, 13]. Another strong predictor of adverse outcomes is the time spent on ECMO. The Viennese group suggests re-evaluating therapeutic strategies after seven days of ECMO support because mortality disproportionally increases afterward [12, 13, 15]. Our patient was younger and had a relatively short duration of ECMO (86 h), which could explain the satisfactory outcome.

One of the most significant problems with ECMO support is the lack of LV unloading. Placing a vent through the right superior pulmonary vein is helpful as the IABP does not substantially unload the LV. Another option is to place an Impella device (Abiomed) either through the femoral artery or subclavian artery to unload the LV in a patient on peripheral ECMO.

Like many low-volume centers, ECMO cannulations are a relatively new procedure that was introduced to our institution ~7 years ago. Initially, perfusionists were on-site 24 h a day; however, with gained experience, our institution’s staff has become more comfortable and considers ECMO a routine support system. Perfusionists check the ECMO circuit every 8 h in person; however, clear and responsive communication between the bedside team and the perfusionists is key.

Our case report adds to the previously reported notion that immediate temporary MCS placement stabilizes these critically ill patients, allows for myocardial maturation before surgical VSD repair, and carries better survival [16]. A precise surgical intervention, vasopressor/inotrope support, temporary MCS (IABP ± ECMO), and timely coordination between the multidisciplinary cardiac and critical care teams could result in patient survival in less experienced centers.

## Data Availability

All available data are incorporated into the article.
